# Therapeutics of the Throat

**Published:** 1890-09

**Authors:** E. B. Nash

**Affiliations:** Cortland, N. Y.


### THERAPEUTICS OF THROAT.

E. B. Nash, M. D., Cortland, N. Y.

In June, 1887, through the pages of The Homœopathic Physician, I proposed a work on therapeutics of the throat, said work to be prepared by members of the I. H. A.

The proposition met with a very kindly reception, and the work is slowly but surely progressing. The chief reason of the delay is that a few competent men whom we are very anxious to have represented, have not yet performed their *promised* work.

Of course, the time of the busy practitioner is not his own always, and, consequently, those who would, have not been able to perform for want of time. So we blame no one.

Again, some, like our lamented Lippe, have been called from their labors to rest. Others still, like Drs. Ballard and Carlton, have been disabled by sickness.

I thought I would "rush" into print once more and let the members know the condition of the work at the present.

The following remedies have been worked and published in Vols. VII, VIII of THE HOMŒOPATHIC PHYSICIAN:

Phytolacca, E. B. Nash.

Belladonna, W. S. Gee.

Æsculus-hip., Ailanthus, } J. V. Allen.

Ignatia, G. H. Clark.

Cistus-can., E. J. Lee.

Dioscorea, Indium, } J. G. Bell.

Arum-tri., W. J. Guernsey.

Baptisia, G. W. Sherbino.

Carb-an., Carb-veg., } Ed. Cranch.

Baptisia, G. W. Sherbino.

Rhus-tox., C. E. Chase.

Sepia, B. L. B. Baylies.

Those that are worked and in my possession, not yet having appeared in print, are

Lachesis, C. C. Smith.

Merc-viv., Merc-sul., Merc-prot., Merc-bin., Merc-cyan., } C. H. Allen.

Causticum, T. D. Stowe.

Alumina, M. Preston.

Conium, Hoopes.

Kreosote, Alice B. Campbell.

Staph., L. R. Thurston.

Bryon., W. H. Baker.

Mag-carb., J. B. G. Custis.

Nat-mur., Nat-ars., Nat-phos., } G. W. Sherbino.

Petrol., J. Schmidt.

Sulph., J. T. Kent.

Arg-met., Arg-nit., } E. Rushmore.

Sabad., Iod., Ferr-phos., } C. F. Nichols.

Lycop., P. P. Wells.

Pulsat., J. A. Biegler.
Apis, Wm. Wesselhœft.
Nux-v., D. C. McLaren.
Silicea, J. C. Roberts.
Acet-acid., J. T. Kent.
Anac., Nathan Cash.
Gelsem., A. McNeil.
Baryta-c., G. W. Sherbino.
Lac-can., E. M. Santee.
Ammon-carb., Ammon-mur., Walter M. James.

Here are fifty remedies already worked.

Those unworked but still promised are :

Aconite, E. J. Lee.
*The Arsenicums, E. H. Ballard.
The Calcareas, the Powells.
The Kalis, C. W. Butler.
Phos., Wm. J. Guernsey.
Cantharis, C. C. Smith.
Capsicum, E. W. Sawyer.
Dulc., A. McNeil.
Nit-acid., E. P. Hussey.
Psorin, W. A. Hawley.
Sul-acid., W. H. Leonard.

Unless some one will volunteer to help out Dr. Ballard, I shall try to do the Arsenicums myself.

I hope to hear very soon from every one of the rest of the names in this list, and please let us know if the I. H. A. may yet depend on you for your part of this work.

There are a few other remedies that ought to come out, viz.:

| | | |
|---|---|---|
| Syphilinum, | Hepar-sul., | Medorrhinum, |
| Guai., | Spongia, | Hydrastis, |
| Mezer., | Wyethia, | Mur-ac. |
| Hydrophobinum, | | |

Who will volunteer to arrange these?

When these remedies all worked come in, it is my purpose to notify each member to cast his vote as to how and where all the remedies shall be published, each member being entitled to one vote for each remedy he has worked. Now, on the "home-stretch," let every man push, or else "pull out" of the work and give some one else his place.

My own idea is that these remedies should be published as an

* Dr. Ballard notified me that on account of ill-health he would be unable to finish his work, much as he would like to.

appendix to some journal and then the Reportory arranged afterward, but this is for the members of the I. H. A. to decide.

The remedies, as may be seen by the published ones in THE HOMŒOPATHIC PHYSICIAN, are not all worked according to the plan which I recommended in my arrangement of Phytolacca. Indeed, others have improved upon it, but I would suggest to let every one's work go in as he prepared and signed his name to it, and a Repertory can be arranged from it in that way just as well.

In conclusion, if I have left out any one, or any remedies, or have made any omissions or mistakes which need corrections, remember corrections or suggestions are always in order.

